# The Transcriptomic Basis of Oviposition Behaviour in the Parasitoid Wasp *Nasonia vitripennis*

**DOI:** 10.1371/journal.pone.0068608

**Published:** 2013-07-19

**Authors:** Bart A. Pannebakker, Urmi Trivedi, Mark A. Blaxter, Rebekah Watt, David M. Shuker

**Affiliations:** 1 Laboratory of Genetics, Wageningen University, Wageningen, The Netherlands; 2 The Institute of Evolutionary Biology, School of Biological Sciences, University of Edinburgh, Edinburgh, United Kingdom; 3 The School of Biology, University of St Andrews, St Andrews, United Kingdom; Duke University, United States of America

## Abstract

Linking behavioural phenotypes to their underlying genotypes is crucial for uncovering the mechanisms that underpin behaviour and for understanding the origins and maintenance of genetic variation in behaviour. Recently, interest has begun to focus on the transcriptome as a route for identifying genes and gene pathways associated with behaviour. For many behavioural traits studied at the phenotypic level, we have little or no idea of where to start searching for “candidate” genes: the transcriptome provides such a starting point. Here we consider transcriptomic changes associated with oviposition in the parasitoid wasp *Nasonia vitripennis*. Oviposition is a key behaviour for parasitoids, as females are faced with a variety of decisions that will impact offspring fitness. These include choosing between hosts of differing quality, as well as making decisions regarding clutch size and offspring sex ratio. We compared the whole-body transcriptomes of resting or ovipositing female *Nasonia* using a “DeepSAGE” gene expression approach on the Illumina sequencing platform. We identified 332 tags that were significantly differentially expressed between the two treatments, with 77% of the changes associated with greater expression in resting females. Oviposition therefore appears to focus gene expression away from a number of physiological processes, with gene ontologies suggesting that aspects of metabolism may be down-regulated during egg-laying. Nine of the most abundant differentially expressed tags showed greater expression in ovipositing females though, including the genes *purity-of-essence* (associated with behavioural phenotypes in *Drosophila*) and *glucose dehydrogenase* (*GLD*). The GLD protein has been implicated in sperm storage and release in *Drosophila* and so provides a possible candidate for the control of sex allocation by female *Nasonia* during oviposition. Oviposition in *Nasonia* therefore clearly modifies the transcriptome, providing a starting point for the genetic dissection of oviposition.

## Introduction

A key challenge facing biologists is to link the genotype to the phenotype [1]. For behaviour this is a particular challenge, as behaviours may be complex, highly environment-dependent phenotypes [2]–[4]. The rationale for trying to understand the genotype-phenotype map is either mechanistic (how are organisms made?) or functional (what genes generate the variation that evolution acts upon?). Traditional approaches to this problem have either worked from the bottom-up, exploring the phenotypic consequences of mutants to elucidate the pathways that generate phenotypes up the hierarchy of biological organisation (from cells, to tissues, to whole organisms), or instead they have worked from the top-down, seeking to describe whole organism variation in terms of genes or genomic regions. Both approaches have proved successful (for textbook treatments see [5]–[8]). However, it has become clear that, despite this success, we need to bridge the gap between these two approaches more completely if we are to map DNA sequences onto phenotypic variation, and thus link the molecular and phenotypic evolution of behaviour [9], [10].

Part of the problem has been that early optimism regarding the genetic basis of behaviour has been confounded by the number (and to some extent the identity) of genes associated with behavioural phenotypes [11]. In some fortunate cases, one or a few known genes do appear to provide a good understanding of the genetic basis of a given behaviour, and naturally-occurring variation in those genes has been identified. Perhaps two of the best examples involve the rover-sitter polymorphism in *Drosophila melanogaster* larvae that influences foraging strategies and length of foraging trails (associated with the *foraging* (*for*) gene, now known to be a cGMP-dependent protein kinase [12], and also the mechanism underlying circadian behavioural rhythms, again in *Drosophila* (associated with genes such as *period* and *timeless* [13]). However, these apparently simple single- or few-gene examples are rare and perhaps more complex than originally thought. It is becoming increasingly clear that many hundreds of sequences, coding and non-coding, may act in concert to influence behavioural phenotypes, and that individual mutations may vary in their phenotypic effects depending upon the genetic background (for instance outside of the standard laboratory genetic backgrounds of model organisms [11]). Indeed, despite the success of quantitative trait loci (QTL) studies in identifying genomic regions associated with many traits [14], including complex behaviour (such as sex allocation [15]), more recent work has confirmed that QTL or genome-wide association studies (GWAS) can give an unrealistically simple view of genetic architecture (for critiques see [9], [16]). This is exemplified by the “missing heritability” problem [17]. Even when a gene is found to be associated with a particular behaviour it may be a highly upstream gene with many pleiotropic effects (e.g. *fruitless* in *Drosophila melanogaster* [18]). Mutations of such genes may yield behavioural pathologies, but it remains an open question as to whether such loci, or their regulatory sequences, typically contribute to the segregating variation in natural populations of interest to evolutionary biologists.

Recent developments in microarray and sequencing technologies have offered an alternative approach that may help fill this gap: the large-scale analysis of the genes actually expressed in cells or tissues, or during key periods of development or behaviour [3], [4], [19]–[23]. These transcriptomic approaches offer a means of directly exploring which genes are associated with complex phenotypes such as behaviour, and experiments can reveal changes in gene regulation associated with changes in those phenotypes. For instance, recent work has revealed some of the genes and genetic networks associated with changes in female behaviour after mating in species of *Drosophila* [24]–[30], the Mediterranean fruit fly *Ceratitis capitata* [31], the honey bee *Apis mellifera* [32], [33], and the parasitoid wasp *Nasonia vitripennis* (R. Watt, U. Trivedi, T.J. Park, M. Blaxter and D.M. Shuker, unpublished data). These data may help identify physiological and neurological mechanisms associated with behaviours, and thus “candidate genes”, which is important as many of the behaviours studied by behavioural ecologists may be far removed from the often simpler behaviours that are studied in behaviour genetics laboratories. However, an animal’s behavioural repertoire is also influenced by the existing neural architecture and its physiology, such as titres of hormones and other signalling molecules, and so it remains an open question as to whether changes in gene expression contemporaneous to the performing of a behavioural act can meaningfully identify genes associated with that behaviour.

Here we consider whether a transcriptomic approach can reveal changes in gene expression during oviposition in the parasitoid wasp *Nasonia vitripennis*. Oviposition is a fundamental physiological process for females that need to invest limited resources into the production and maturation of eggs, which could otherwise have been invested in other somatic functions [34]. Furthermore, oviposition is a crucial reproductive decision that females of many species have to make. In terms of insects such as parasitoid wasps, oviposition is a key determinant of larval success and is the only form of parental care a female will provide for her offspring [35]. For *Nasonia*, females have to find and choose suitable hosts (blowfly pupae of the appropriate species, quality and developmental stage). They then have to decide how many eggs to lay (*Nasonia* are gregarious, laying multiple eggs per host puparium), and what sex ratio to produce. There is substantial evidence from *Nasonia* that females use an array of cues when finding, choosing and utilising hosts for egg-laying [36]–[40]. However, our understanding of the genetics underlying these important processes and decisions remains very limited [15], [34], [41], [42].

Importantly, a fully sequenced genome is available for *Nasonia vitripennis* and two of its con-geners (*N*. *longicornis* and *N*. *giraulti*), providing a starting point for genomic analyses of complex traits [43]. We have taken advantage of the availability of whole-genome information and associated gene annotations to explore what genes are expressed by female *Nasonia vitripennis* during oviposition. Insight into the genetic background of parasitoid oviposition will be crucial to understand how evolution acts upon a this fundamental physiological and behavoural trait. To the best of our knowledge, this is the first description of the oviposition transcriptome for any parastioid wasp.

## Materials and Methods

### Study organism

*Nasonia vitripennis* (Hymenoptera: Chalcidoidea) is a generalist gregarious wasp that parasitises large dipteran pupae (including Sarcophagidae and Calliphoridae [43], [44]). Depending on the host species, females oviposit between 20–50 eggs on an individual host, with male offspring emerging just before females (after approximately 14 days at 25°C). Females are synovigenic, i.e. they are born with a limited number of mature eggs in their ovarioles, but can produce and mature further eggs provided they have protein resources available [45]. Males have small wings and are unable to fly, remaining close to the emergence patch where they compete with each other for matings with emerging females. Females are fully winged and typically mate only once before dispersing to find new hosts.

The wasps used in this experiment were from the AsymC strain, which was isolated in 1986 by curing the wild-type strain LabII of *Wolbachia* [46]. Wasps have been maintained on *Calliphora vomitoria* or *C*. *vicina* hosts at 25°C, 16L: 8D light conditions since this time, allowing AsymC to become highly inbred. The AsymC line was sequenced and annotated in the *Nasonia* genome project [43], allowing for the direct mapping of our transcriptomic data on to the available genomic resources.

### Oviposition experiment

In order to control for possible host and genotype effects, we isolated a single 2-day old mated AsymC female in a glass vial and provided her with a single host to produce F1 daughters. Eight 2-day old mated F1 females were subsequently provided with three hosts to produce the F2 test females. We randomly selected one host from each F1 female, and isolated 16 2-day old mated F2 test females in glass tubes, of which eight were randomly allocated to the oviposition treatment and eight to the resting treatment. We provided the test females with a single host for 24 hours as pre-treatment to facilitate egg development [45]. We then discarded the pre-treatment hosts and gave each female a piece of chromatography paper soaked in honey solution for a further 24 hours.

For the experiment, we transferred the females to 1.5 mL Eppendorf tubes that contained a single host for the oviposition experiment, or were empty for the resting treatment. After 60 mins, females were flash-frozen in liquid nitrogen and stored on dry-ice until the addition of RNAlater-ICE (Ambion, Austin, TX, USA) after which they were transferred to −20^o^C. All females in the oviposition treatment were observed to have commenced ovipositing. We pooled the F2 test females from each F1 mother according to treatment, generating a total of eight pooled samples per treatment (consisting of 8 females per pool) for RNA isolation and sequencing (i.e. N = 16 for the whole experiment).

### RNA isolation

RNA was isolated using Qiagen RNeasy Mini Kit (Qiagen, Valencia, CA, USA) according to the manufacturer’s protocol. We further purified our samples using Turbo DNase (Ambion, Austin, TX, USA) and tested the integrity and concentration of our samples using a NanoDrop spectrophotometer (Nanodrop Technologies, Wilmington, DE, USA).

### Digital gene expression tag profiling (DGE)

Tag library preparation was done using the DGE-Tag Profiling for *Nla*III Sample Prep kit (Illumina, San Diego, CA, USA) according to the manufacturer’s protocol. In short, mRNA was first captured from the total RNA by magnetic oligo(dT)beads. mRNA was then converted into cDNA and bead-bound cDNA was subsequently digested with *Nla*III. Fragments other than the 3' cDNA fragments attached the beads were washed away, and a *Nla*III-adapter containing a *Mme*I recognition site was ligated to the 5' end of the bead-bound cDNA. *Mme*I cuts to 21bp downstream from its recognition site, creating 21bp tags that start with the *Nla*III recognition site, CATG. A second adapter was then ligated at the *Mme*I site, allowing for PCR amplification, followed by attachment of the enriched tags to the surface of the sequencing flowcell in which they were sequenced by synthesis using the Illumina Genome Analyzer I system at GenePool, the University of Edinburgh's in-house sequencing facility. The raw data (tag sequences and counts) were deposited in NCBI's Gene Expression Omnibus and are accessible through GEO Series accession number GSE43352 (http://www.ncbi.nlm.nih.gov/geo/query/acc.cgi?acc=GSE43352).

### DGE tag mapping and filtering

Reads were first aligned against the *Nasonia vitripennis* genome (Nvit1.0, ftp://ftp.hgsc.bcm.tmc.edu/pub/data/Nvitripennis/fasta/Nvit_1.0/linearized_sequence) using MAQ 0.6.8 [47], allowing for a 2bp mismatch between the 21bp tag and reference sequence. Only tags with a phred mapping quality threshold of 30 were used in further analysis, corresponding to one wrongly aligned sequence in 1000. We then aligned the remaining tags to the *Nasonia* Official Gene Set (OGS) v.1.2 (http://hymenopteragenome.org/Nasonia/) using the same mapping settings. Tags that failed to map to the *Nasonia* genome or the OGS were classed as null and not considered in further analysis. Whilst DGE tag profiling produces both sense and antisense tags, the majority of antisense tags produced in our set-up are most likely the result of technical artefacts [48]. We therefore deemed it appropriate to remove all antisense tags from our analysis. Transcripts present at extremely low abundance are a common source of noise in these kinds of studies and likely include technical artefacts. We therefore also excluded tags that had 15 or less reads across all 16 samples (i.e. a mean of less than one read per replicate). In terms of the dataset, we initially had 74,173,884 reads comprising 215,130 tags. Following removal of anti-sense and null sequences we had 32,036,887 reads from 88,627 tags (Table S1). Removing the very low abundance transcripts left us with 31,807,132 reads from 30,334 tags for our analysis (i.e. the last step removed 65.8% of tags but only 0.7% of reads).

### Statistical analysis

We tested for differential expression between ovipositing and resting females using DESeq v2.10 [49], run with R version 2.15.0 [50]. Briefly, DESeq employs a negative binomial error distribution to model transcript abundance in high-throughput datasets. With the comparatively high level of replication (for this kind of study) used in this experiment (N = 8 replicates per treatment), we used empirical estimates of the deviance of tag counts for each tag (using the option “gene-est-only”). DESeq estimates the significance of differential expression for each tag, and then corrects for the multiple comparisons using the false discovery rate (FDR) correction of Benjamini & Hochberg [51]. We considered tags to be differentially expressed if they had an adjusted p-value after FDR correction of p<0.1. We also report which of these tags that have greater than 4-fold log2 gene expression differences (another common measure of differential expression).

### Annotation and Gene Ontology

We annotated our tags using the *Nasonia* Official Gene Set version 2 (OGS2, January 2012, http://arthropods.eugenes.org/genes2/Nasonia/). Briefly, annotation for the tags aligned to the genome was done on the basis of their genomic position, with tags that were first aligned to the OGS1.2 converted to OGS2 based on gene equivalence. In total 25,427 out of 30,344 (83.8%) tags were annotated with the OGS2. Each tag was then assigned gene ontology (GO) terms using the *Nasonia* GO annotation for the OGS2 generated as part of an effort by the *Nasonia* community (http://hymenopteragenome.org/Nasonia/?q=evidential_gene_data). Singular enrichment analysis of the GO terms was performed in agriGO (http://bioinfo.cau.edu.cn/agriGO [52]) using chi-square tests to compare differentially expressed genes to the *Nasonia* GO annotation.

## Results

**Figure 1:**
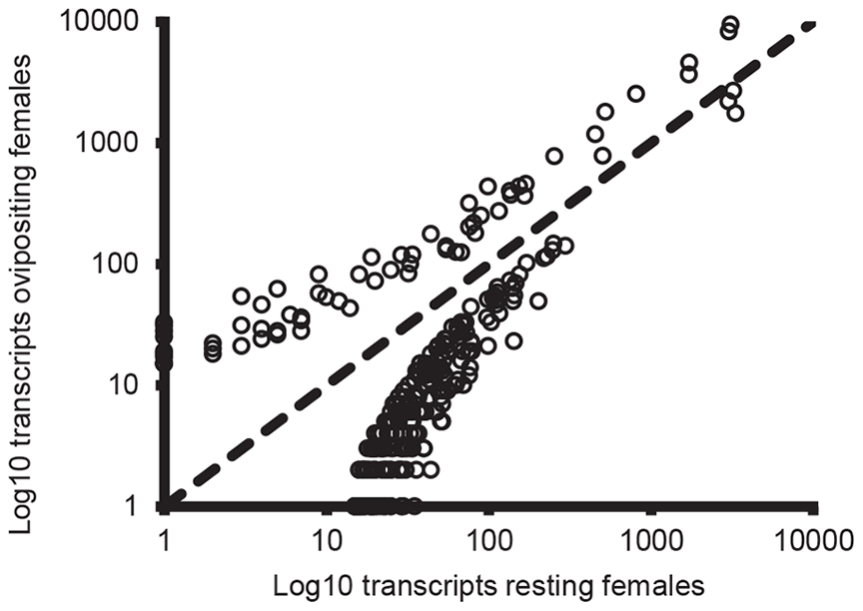
Differentially expressed tags between resting and ovipositing *Nasonia vitripennis* females. Total transcript abundance by treatment for the 322 tags differentially expressed between resting and ovipositing *Nasonia vitripennis* females (summed across N = 8 biological replicates per treatment). Approximately 77% of these tags show higher expression levels in resting females. The dotted line indicates a 1:1 relationship. All tags with total abundance across both treatments of <16 counts were excluded from the analysis.

Oviposition in *N. vitripennis* females is associated with changes in gene expression at the whole-body level. From a total of 30,334 tags, 322 showed significant differential expression at a FDR of 0.1 (unadjusted p-values: all p<0.0012), with 209 significant at a FDR of 0.05 (Table S2). These differentially expressed tags represent 84,326 reads out of a total of 31,807,132, or 0.27% of the total transcriptome. Oviposition appears to be associated with a focusing of gene expression: 73 tags (22.7%) show significantly greater expression in ovipositing females, whilst 249 (77.3%) show greater expression in resting females (Figure 1). In addition, 9 of the 12 differentially expressed tags with total counts across all replicates in excess of 1000 are associated with greater expression in ovipositing females (see also Table 1). Forty-four tags (13.7% of our differentially expressed tags) had a greater than 4-fold log2 change in expression between treatments (Table 2). Six of these tags showed higher expression in ovipositing females, whilst the other 38 tags showed higher expression in resting females.

**Table 1:** Differentially expressed genes with over 1000 tags in ovipositing versus resting *Nasonia vitripennis* females.

TagID^a^	OGS2 ID^b^	OGS2 Description^c^	Total tag count	log2-fold change	P-value	FDR-adjusted P-value
*Down-regulated*						
65548	Nasvi2EG014964	pontin protein	5902	−0.28	1.867E-04	0.0352
15723	Nasvi2EG020753	Unknown	5207	−0.43	1.049E-03	0.0995
39537	Nasvi2EG010910	Pyrroline-5-carboxylate reductase	5081	−1.19	6.005E-04	0.0695
*Up-regulated*						
70106	Nasvi2EG015670	Glucose dehydrogenase	12589	1.24	5.458E-05	0.0161
25917	Nasvi2EG009277	Unknown	11312	1.27	8.682E-06	0.0050
3864	Nasvi2EG000856	purity of essence protein	6283	1.40	8.996E-05	0.0212
32349	Nasvi2EG001535	Serine/threonine-protein kinase SNF1 kinase 2	5360	0.89	1.285E-05	0.0060
43242	Nasvi2EG025132	Scavenger receptor class B member 1	3344	1.26	3.154E-05	0.0113
84658	Unannotated	-	2324	1.56	2.774E-06	0.0022
9450	Nasvi2EG006942	bcl-2-related ovarian killer protein homolog A	1625	1.28	2.682E-06	0.0022
25434	Nasvi2EG009166	acyl-CoA Delta(11) desaturase. putative	1282	0.58	6.389E-04	0.0723
78621	Nasvi2EG005112	Unknown	1024	1.46	4.024E-06	0.0028

a Tag identifier.

b *Nasonia* Official Gene Set version 2 identifier.

c *Nasonia* Official Gene Set version 2 description.

**Table 2:** Differentially expressed genes in ovipositing versus resting *Nasonia vitripennis* females showing a log2-fold change>4. Where a tag is associated with more than one gene, all genes are given.

	TagID^a^	OGS2 ID^b^	OGS2 Description^c^	log2-fold change	P-value	FDR-adjusted P-value
*Down-regulated*						
	67418	Nasvi2EG003738	Nuclear pore complex protein Nup214	−5.63	6.26E-04	0.0715
		Nasvi2EG003739	WD repeat-containing protein 46. putative			
	44939	Nasvi2EG011935	neutral sphingomyelinase. putative	−5.55	1.54E-05	0.0068
	9088	Nasvi2EG006879	Reverse transcriptase. putative	−5.55	3.97E-04	0.0543
	26758	Nasvi2EG009337	Tyrosine-protein kinase receptor	−5.46	1.80E-06	0.0017
	26588	Nasvi2EG022804	Receptor-interacting serine/threonine-protein kinase 5	−5.26	1.51E-06	0.0016
	24884	Nasvi2EG009056	DNA mismatch repair protein Msh6. putative	−5.11	4.55E-04	0.0593
	37017	Nasvi2EG010367	plexin-A4	−5.09	3.83E-07	0.0006
	17824	Nasvi2EG007903b	UPF0558 protein C1orf156 protein	−5.07	2.29E-05	0.0088
		Nasvi2EG007831	E3 ubiquitin-protein ligase HUWE1			
		Nasvi2EG007910b	pyridoxine-5'-phosphate oxidase			
	74026	Nasvi2EG004564	Unknown	−5.04	2.98E-06	0.0022
		Nasvi2EG004562	Unknown			
	42453	Nasvi2EG024978	Vacuolar protein sorting-associated protein 13B	−4.91	2.29E-05	0.0088
	5216	Nasvi2EG001148	Translation initiation factor eIF-2B subunit epsilon	−4.89	1.73E-04	0.0335
	50462	Nasvi2EG002884	maelstrom. protein	−4.87	4.50E-05	0.0146
	50144	Nasvi2EG002829	RING finger protein 10. putative	−4.80	1.72E-04	0.0335
	17600	Nasvi2EG007832	Sodium-independent sulfate anion transporter	−4.78	4.94E-04	0.0622
		Nasvi2EG007831	E3 ubiquitin-protein ligase HUWE1			
	50230	Nasvi2EG002845	BTB/POZ domain-containing adapter for CUL3-mediated RhoA degradation protein	−4.73	6.80E-04	0.0754
	57086	Nasvi2EG013710	ADIPOR receptor CG5315-like. putative	−4.72	2.54E-04	0.0421
	25366	Unannotated	-	−4.71	4.96E-04	0.0622
	67839	Nasvi2EG003841	Sorting nexin-29	−4.70	7.46E-04	0.0809
	72017	Nasvi2EG004081	NAD-dependent ADP-ribosyltransferase sirtuin-4	−4.69	8.13E-05	0.0206
	59624	Nasvi2EG003310	Metallophosphoesterase 1	−4.68	3.34E-04	0.0490
	33547	Unannotated	-	−4.67	1.16E-05	0.0057
	50943	Nasvi2EG037181	nucleosome assembly protein 1. putative sym:LOC100117842 (100%e)	−4.64	8.81E-05	0.0211
		Nasvi2EG012490	Unknown			
	87355	Nasvi2EG010438	metastasis-associated protein MTA1	−4.55	8.81E-05	0.0211
	63918	Nasvi2EG014482	Nuclear factor related to kappa-B-binding protein	−4.54	6.36E-07	0.0008
	80523	Nasvi2EG005241	sulfide quinone reductase	−4.39	4.64E-04	0.0597
	29205	Nasvi2EG009775	Nodal modulator 2	−4.37	7.32E-04	0.0798
	85289	Nasvi2EG006085	1-acyl-sn-glycerol-3-phosphate acyltransferase gamma	−4.31	4.30E-04	0.0573
		Nasvi2EG006112	archease protein			
	29500	Nasvi2EG009850	prostaglandin reductase 1	−4.31	3.35E-04	0.0490
	82291	Nasvi2EG017861	roadkill protein. putative	−4.31	2.04E-04	0.0377
	76776	Nasvi2EG004717	Solute carrier family 35 member E1	−4.30	1.72E-04	0.0335
	47013	Nasvi2EG012287	ATP-dependent RNA helicase DDX54	−4.30	9.23E-04	0.0920
	34392	Unannotated	-	−4.28	1.28E-07	0.0002
	64874	Nasvi2EG014819	dystrobrevin beta	−4.24	2.31E-04	0.0402
	37450	Nasvi2EG010464	Arrestin domain-containing protein 2	−4.11	2.27E-05	0.0088
	28878	Nasvi2EG023174	henna protein. putative	−4.10	4.70E-08	0.0001
	42342	Nasvi2EG011482	CDP-diacylglycerol-glycerol-3-phosphate 3-phosphatidyltransferase. mitochondrial	−4.10	7.82E-05	0.0202
	14695	Nasvi2EG007438	Unknown	−4.07	1.92E-05	0.0077
	14622	Nasvi2EG007419	Serine/threonine-protein phosphatase 2A regulatory subunit B'' subunit alpha	−4.04	3.25E-04	0.0490
	14622	Nasvi2EG007419	Serine/threonine-protein phosphatase 2A regulatory subunit B'' subunit alpha	−4.04	3.25E-04	0.0490
*Up-regulated*						
	38212	Nasvi2EG010660	Mediator of RNA polymerase II transcription subunit	4.12	1.27E-04	0.0272
	34092	Nasvi2EG002034	neurocalcin homolog	4.17	2.35E-04	0.0404
	46291	Nasvi2EG012198	Unknown	4.67	7.29E-05	0.0194
	81088	Unannotated	-	4.78	6.88E-05	0.0188
	84647	Nasvi2EG005959	Unknown	5.19	5.00E-07	0.0008
	85043	Nasvi2EG006037	ubiquinone biosynthesis protein COQ7	5.37	5.44E-04	0.0647

a Tag identifier.

b *Nasonia* Official Gene Set version 2 identifier.

c *Nasonia* Official Gene Set version 2 description.

The most abundant tag of all mapped to the gene *elongation factor 1-alpha 1* (with an average abundance across all replicates of approximately 60080 reads) which was reassuringly not differentially expressed between our treatments (this gene, with its universal role in translation, is typically used a control for quantitative-PCR studies of differential expression of individual loci for this reason). In total, 17 tags had average abundances in excess of 10,000 reads per replicate. Our most abundant differentially expressed tag mapped to the *glucose dehydrogenase* gene, and had an average abundance of 787 reads per replicate. Forty differentially expressed tags were unique to one treatment or another (32 unique to resting females and 8 unique to ovipositing females), but all of these tags had total counts between 16 (our minimum threshold abundance) and 37.

From the total tag pool of 30,334 tags, 25,427 (83.8%) were annotated, covering 9,182 unique genes in the *Nasonia* OGS2 (37.4% out of total 24,525 genes in OGS2). 4,907 tags (16.2%) did not match any genes in the OGS2. Of the 322 tags showing differentially expression, 286 (88.8%) were annotated, while 36 (11.2%) did not match any OGS2 genes. Of the annotated differentially expressed tags, 163 (57.0%) could be assigned a Gene Ontology (GO) classification representing 38 unique GO terms for the up-regulated tags, and 134 unique GO terms for the down-regulated tags (Figures S1 and S2). Singular Enrichment Analysis of the associated GO terms revealed an overrepresentation of 5 GO terms in the down-regulated tags, all of which related to metabolic processes (Table 3).

**Table 3:** Gene Ontology (GO) terms significantly enriched among differentially expressed tags down-regulated in oviposting versus resting *Nasonia vitripennis* females.

GO term	GO description	Number in down- regulated tag set	Total GO terms in down- regulated tag set	Number in OGS2	Total GO terms in OGS2	P-value	FDR-adjusted P-value
GO:0043436	oxoacid metabolic process	11	134	176	7307	<0.0001	0.0037
GO:0006082	organic acid metabolic process	11	134	176	7307	<0.0001	0.0037
GO:0019752	carboxylic acid metabolic process	11	134	176	7307	<0.0001	0.0037
GO:0042180	cellular ketone metabolic process	11	134	189	7307	0.0002	0.0079
GO:0006520	cellular amino acid metabolic process	7	134	113	7307	0.0027	0.084

## Discussion

Our results show that oviposition is associated with changes in whole-body patterns of gene expression in female *Nasonia vitripennis* wasps. Approximately three-quarters of the changes involve greater expression in resting females (or put another way, down-regulation in ovipositing females), and our enrichment analysis suggests that these down-regulated genes are more likely to be involved in various metabolic processes than expected by chance (Table 3). We therefore suggest that as female wasps move from resting to ovipositing, aspects of their metabolism are down-regulated, focusing gene expression on other processes. Similar gene expression focusing has been reported in *Drosophila* responding to environmental stress, where metabolism related genes are down-regulated and only few stimulus specific stress genes are up-regulated [53]. However, from the outset we note that the changes in gene expression we have uncovered represent less than half of one percent of the total (whole-body) transcriptome, so that at least at this level of resolution many genes are unaffected by the switch in behaviour. At such an early stage in the development of the field of behavioural transcriptomics it is unclear how common a finding this will be. Of course, many physiological functions have to carry on regardless of what an animal is doing, and all of our most highly expressed tags were not differentially expressed (indeed, the top 362 tags in terms of average counts were not differentially expressed).

Alongside the key result that oviposition is associated with broad-scale changes in gene expression, with metabolic processes affected more than expected by chance, to what extent have we been able to identify likely candidate genes that influence oviposition behaviour? Nine of our most abundant differentially expressed tags showed greater levels of expression in ovipositing females (Table 1). These included the gene *purity-of-essence* (*poe*), which is also known as *pushover* (*push*) in *Drosophila melanogaster* [54]. The purity-of-essence protein is an evolutionarily conserved, large membrane protein containing two zinc finger domains (a structural protein motif associated with binding DNA, RNA, or proteins [55]) that influences behaviour and synaptic transmission in *D*. *melanogaster*. Two mutants of *poe*/*push* in *Drosophila* both cause increased nervous excitability and reduced motor function [54] and mutants also influence peripheral nerve morphology [55]. The gene potentially has other functions (perhaps associated with synaptic transmission), with mutants being associated with sterility in male *Drosophila* [54], and an incomplete *push*/*poe* protein has also been identified as a calmodulin-binding protein expressed in fly photoreceptors [56]. This is perhaps our best candidate for a gene that might influence the motor control associated with handling hosts and/or the resulting drilling into the host puparium, followed by the mechanical control of egg laying.

Also in this group of up-regulated genes is *glucose dehydrogenase*, the protein of which is associated with successful sperm uptake and release in *Drosophila melanogaster* [57]. This was the most abundant gene that was differentially expressed in our experiment. Whilst in our experiment the resting females were mated and so also had sperm present in their spermatheca, our finding is suggestive that *glucose dehydrogenase* is needed during oviposition for the successful release of sperm. As female *Nasonia* need to control sperm release very precisely in order to allocate sex (with fertilised ova developing into daughters, and unfertilised ova developing into sons), variation in the expression of the *glucose dehydrogenase* gene may contribute to phenotypic variation in sex ratio and constrain a female’s ability to adaptively allocate sex as predicted by Hamilton’s theory of Local Mate Competition (LMC [58]; see also [59]–[61]). As the focus of much of the behavioural work associated with oviposition in *Nasonia* is associated with sex allocation, this will be an extremely interesting gene to consider in more detail, and we are currently exploring changes in gene expression in *Nasonia vitripennis* females when we experimentally manipulate the cues females use to make their sex allocation decisions.

Another encouraging candidate is found amongst the 44 of our tags that exhibited greater than 4-fold log2 differential expression (13.7% of our DE tags). Six of these showed greater levels of expression in ovipositing females, including a neurocalcin homolog. The neurocalcin protein is a neuronal calcium-binding protein that may be involved in neurotransmitter release and the regulation of neural function [62]. In vertebrates, *neurocalcin* may be important for regulating sexual dimorphism of the neural song system in birds [63] and sexually dimorphic patterns of expression have also been shown in rodents [64]. Whilst the possible functions of neurocalcin-like proteins are poorly known in insects, a *neurocalcin* is expressed in *Drosophila* neurones [65] and as such this is again the kind of gene we would expect to show changes in the pattern of expression during a behaviour in which females had to either neurally process information and/or perform a complex set of motor patterns.

Otherwise, the links to behaviour are less immediate. For instance, we found that the genes *Scavenger receptor class B member 1* (*SR-B1)* and *acyl-CoA delta-11-desaturase* were up-regulated in ovipositing females. These genes code for proteins associated in lipid transport and fatty acid metabolism [66], [67], and so they may influence how lipids are mobilised during oviposition either in terms of releasing energy reserves for the behaviours themselves or in terms of energy reserves needed for future oogenesis to replace the eggs being deposited. The gene *bcl-2-related ovarian killer* (*BOK)* was also up-regulated during oviposition. *BOK* homologues regulate programmed cell death (apoptosis) during *Drosophila* oogenesis [68], which perhaps suggests that apoptosis is occurring during oviposition in *Nasonia*. Other up-regulated genes include a ubiquinone biosynthesis gene, which again is associated with energy production via the electron transport chain [69]. All these latter cases are perhaps better interpreted as changes that result from the physiological mechanisms involved in oviposition (for instance in terms of the organismal and life-history changes associated with oviposition and the energy utilised during egg-laying), rather than changes that initiate the oviposition behaviours themselves. This fits with the predominant pattern in our data mentioned above, namely the reduction in expression of genes associated with a range of metabolic processes when females are ovipositing. As such, we might be picking up more of the genetic basis of the physiological and life-history consequences of oviposition, and in particular the change in energy-use and metabolism associated with reproductive behaviour, than we are picking up the genetic signals of the behaviour itself. In the very simple experiment undertaken here, disentangling cause and effect for genes and behaviour is clearly difficult, especially with our limited understanding of how cellular processes interact with whole-organism behaviour.

In fact, one of the crucial limitations of behavioural transcriptomics at the moment is that our annotations of genes are associated with molecular functions at a cellular or tissue level that may seem a long way from the regulation or control of a focal behaviour (unless we hit a key developmental gene or signalling pathway). This limitation has recently been articulated by Pavey et al. [70], who suggest that new gene ontologies with explicit ecological (or in our case behavioural) functions might be a useful way to generalise the patterns of associations between molecular and whole-organism phenotypes. For example, it might be that pathways associated with the control of nutrition (such as the insulin-like signalling pathway) end up being implicated in numerous behaviours and life-history decisions because of the fundamental importance of energy acquisition and allocation. These patterns would be easier to recognise if we had more explicit ecological or behavioural designations for genes, built up through studies such as the one we present here. That said, even this will only be a start, as functional analyses in which genes are knocked-down will remain crucial to real progress. Fortunately, techniques such as RNA-interference (RNAi), whereby gene transcripts of focal genes can be targeted and rendered non-functional through cleavage (i.e. transcriptional silencing), are becomingly increasingly available in non-model organisms, including *Nasonia*, crickets, beetles and bugs (e.g. [71]–[77] ).

To conclude, oviposition modifies the pattern of gene expression in female *Nasonia vitripennis*, suggestive of a down-regulation of aspects of metabolism during oviposition behaviour. At the moment we know rather little about the genetic basis of oviposition behaviour in *Nasonia*, or indeed in any other insect. What we do know so far is mostly in terms of genetic variation in oviposition decisions, both in *Nasonia* and elsewhere (e.g. [15], [42], [78], [79] ). Here we have been able to begin the process of identifying putative candidate genes associated with oviposition, including identifying a protein known to influence sperm usage in flies (*glucose dehydrogenase*). However, more generally we hope that our results encourage more animal behaviour researchers to begin to explore how patterns of gene expression are associated with their own behaviours of interest, using the sequencing technologies now readily available. Indeed, currently perhaps the most valuable aspect of studies such as ours is in terms of generating hypotheses about the mechanisms underlying the behaviour we study, hypotheses that can and should be tested comparatively across as many species as possible.

## Supporting Information

Figure S1 Proportion of Gene Ontology (GO) terms for biological process (a), molecular function (b) and cellular component (c) in up-regulated tag set (white bars) and in Official Gene Set 2 (black bars) in ovipositing versus resting *Nasonia vitripennis* females.
(PDF)

Figure S2 Proportion of Gene Ontology (GO) terms for biological process (a), molecular function (b) and cellular component (c) in down-regulated tag set (white bars) and in Official Gene Set 2 (black bars) in ovipositing versus resting *Nasonia vitripennis* females. Significantly enriched terms are indicated with an asterisk.
(PDF)

Table S1 **Valid tags used for analysis of digital gene expression between ovipositing and resting** ***Nasonia vitripennis*** **females.** Table shows tag identifier (TagID), location of tag in *N. vitripennis* genome (Nvit 1.0) or Official Gene Set v1.2 (Location) and tag sequence (TagSequence).
(XLSX)

Table S2 **Differentially expressed tags in ovipositing versus resting** ***Nasonia vitripennis*** **females.** Table shows tag identifier (TagID), tag sequence (TagSequence), mean counts, averaged over all samples from both conditions (baseMean), mean counts from ovipositing females (baseMeanA), mean counts from resting females (baseMeanB), fold change from ovipositing versus resting females (foldChange), the logarithm (to basis 2) of the fold change from ovipositing versus resting females (log2FoldChange), P-value for the statistical signicance of the change in expression (P.value) and P-value adjusted for multiple testing with the Benjamini-Hochberg procedure (FDR.adjusted.P.value).
(XLSX)
