# Correction: The Transcriptomic Basis of Oviposition Behaviour in the Parasitoid Wasp *Nasonia vitripennis*

**DOI:** 10.1371/annotation/820ede3e-65d1-404a-9ddd-4db415ea760e

**Published:** 2013-10-23

**Authors:** Bart A. Pannebakker, Urmi Trivedi, Mark L. Blaxter, Rebekah Watt, David M. Shuker

The name of the third author was incorrectly given. The correct name is: Mark L. Blaxter. The correct citation is: Pannebakker BA, Trivedi U, Blaxter ML, Watt R, Shuker DM (2013) The Transcriptomic Basis of Oviposition Behaviour in the Parasitoid Wasp *Nasonia vitripennis*. PLoS ONE 8(7): e68608. doi:10.1371/journal.pone.0068608. The correct abbreviation in the Author Contributions Statement is: MLB. 

