# Supplementary figures and images for: The Transcriptomic Basis of Oviposition Behaviour in the Parasitoid Wasp Nasonia vitripennis
Source: PLoS One. 2013 Jul 19;8(7):e68608. doi: 10.1371/journal.pone.0068608 (PMC3716692; doi:10.1371/journal.pone.0068608)

**A****Biological process**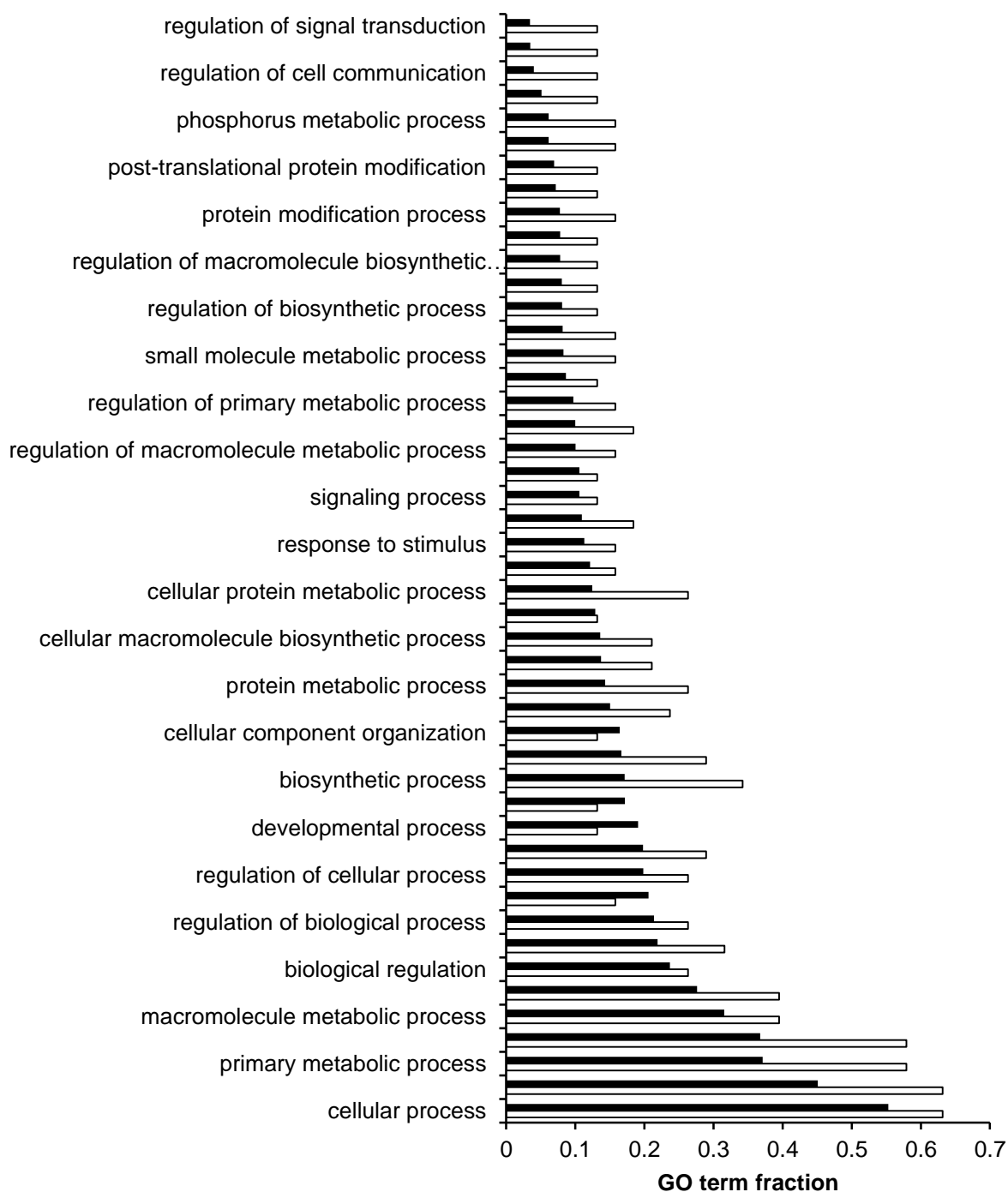

**B**

**Molecular function**

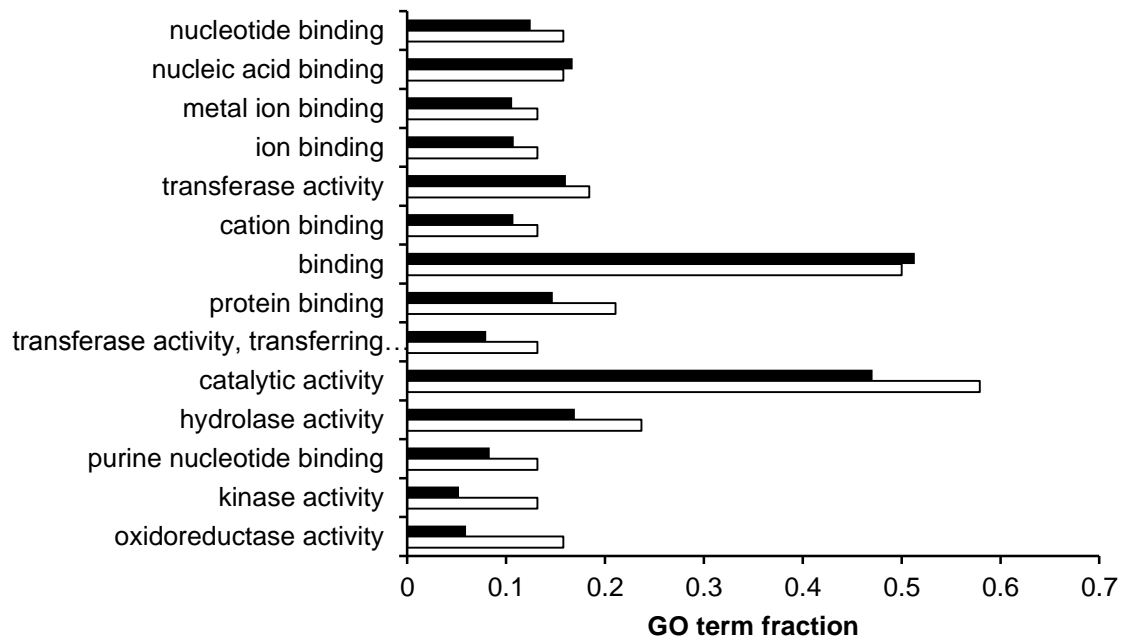

**C**

**Cellular component**

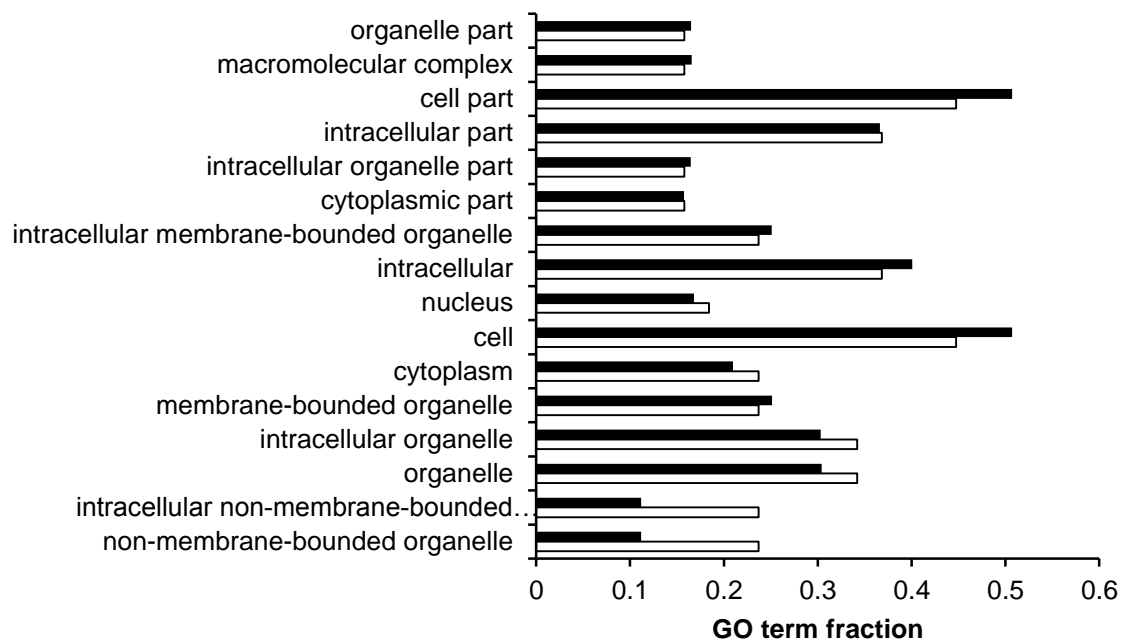

Supplement: Figure S1 — Proportion of Gene Ontology (GO) terms for biological process (a), molecular function (b) and cellular component (c) in up-regulated tag set (white bars) and in Official Gene Set 2 (black bars) in ovipositing versus resting Nasonia vitripennis females. (PDF) [file pone.0068608.s001.pdf]

**A**

## Biological process

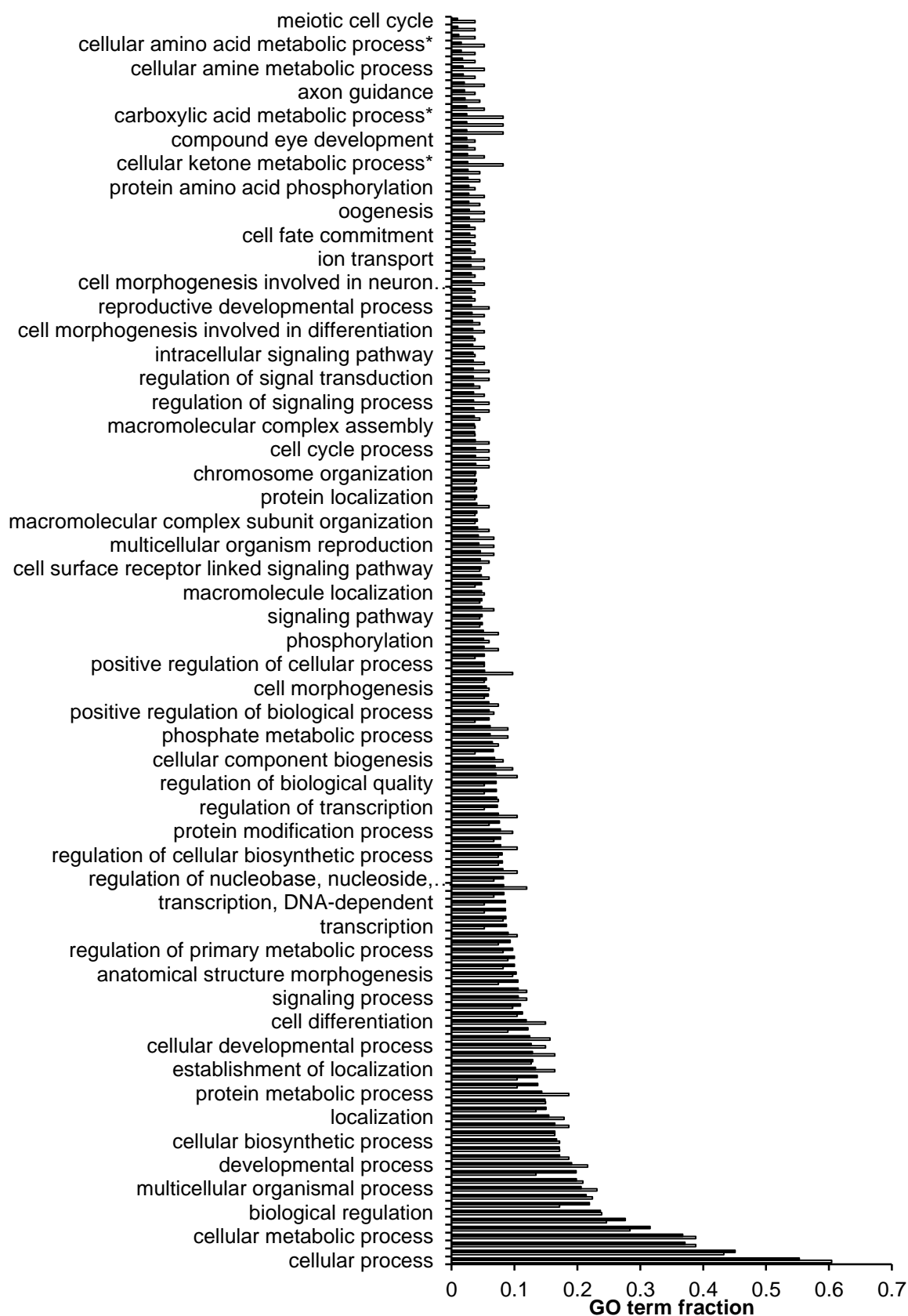

**B****Molecular function**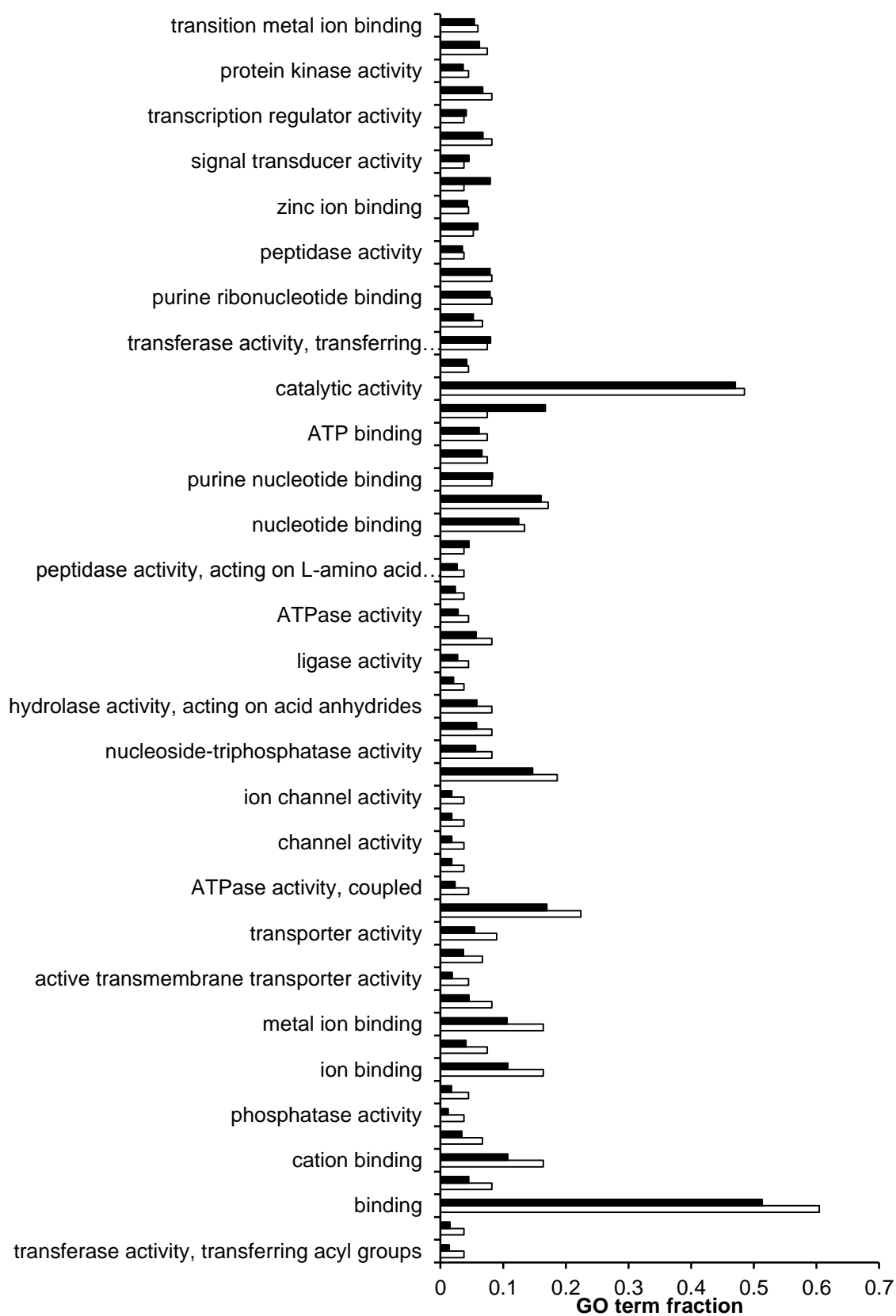

**C****Cellular component**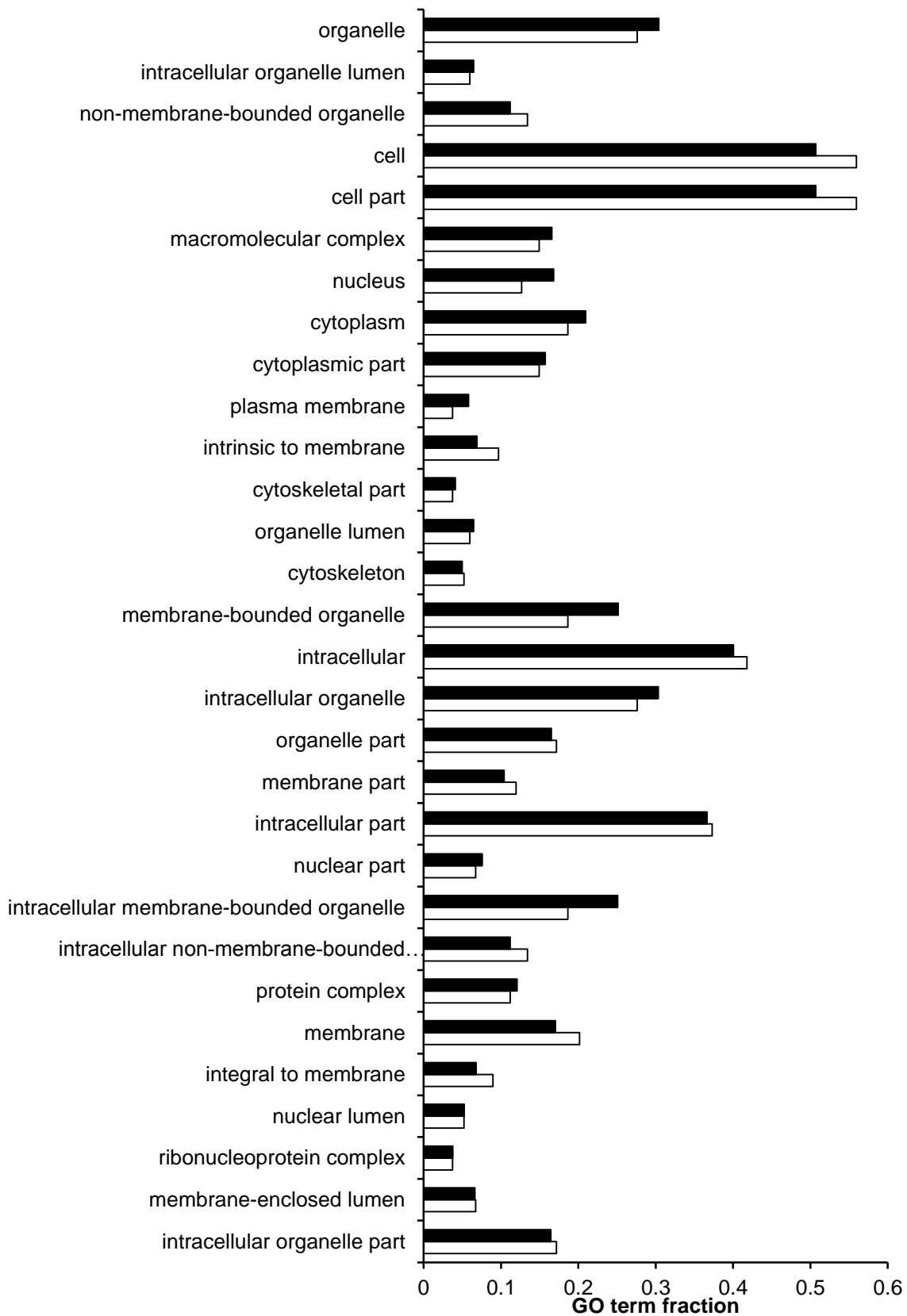

Supplement: Figure S2 — Proportion of Gene Ontology (GO) terms for biological process (a), molecular function (b) and cellular component (c) in down-regulated tag set (white bars) and in Official Gene Set 2 (black bars) in ovipositing versus resting Nasonia vitripennis females. Significantly enriched terms are indicated with an asterisk. (PDF) [file pone.0068608.s002.pdf]
